# Reindeer Anthrax in the Russian Arctic, 2016: Climatic Determinants of the Outbreak and Vaccination Effectiveness

**DOI:** 10.3389/fvets.2021.668420

**Published:** 2021-06-24

**Authors:** Elena A. Liskova, Irina Y. Egorova, Yuri O. Selyaninov, Irina V. Razheva, Nadezhda A. Gladkova, Nadezhda N. Toropova, Olga I. Zakharova, Olga A. Burova, Galina V. Surkova, Svetlana M. Malkhazova, Fedor I. Korennoy, Ivan V. Iashin, Andrei A. Blokhin

**Affiliations:** ^1^Federal Research Center for Virology and Microbiology, Nizhny Novgorod Research Veterinary Institute - Branch of Federal Research Center for Virology and Microbiology, Nizhny Novgorod, Russia; ^2^Federal Research Center for Virology and Microbiology (FRCVM), Pokrov, Russia; ^3^Faculty of Geography, Lomonosov Moscow State University, Moscow, Russia; ^4^FGBI Federal Centre for Animal Health (FGBI ARRIAH), Vladimir, Russia

**Keywords:** anthrax, arctic, climate, outbreak, reindeer, vaccination, Russia, indirect hemagglutination assay

## Abstract

The Yamal Peninsula in the Russian Federation experienced a massive outbreak of anthrax in reindeer (*Rangifer tarandus*) in July–August 2016, with 2,650 (6.46% of the total susceptible population) animals infected, of which 2,350 died (case fatality rate of 88.67%). In our study, we analyzed climatic and epidemiological factors that could have triggered the outbreak. The cancelation of reindeer vaccination against anthrax in 2007 resulted in an increase in population susceptibility. In response to the outbreak, total vaccination of all susceptible animals was resumed. To assess the vaccination effectiveness, we tested 913 samples of blood serum taken from vaccinated reindeer using an antigenic erythrocyte diagnostic kit to detect specific anti-anthrax antibodies via an indirect hemagglutination assay (IHA) 9 months after vaccination. We found that 814 samples had sufficiently high levels of anti-anthrax antibodies to indicate a protection level of 89% (95% confidence interval: 87–91%) of the whole reindeer population. Abnormally high ambient temperature in the summer of 2016 contributed to the thawing of permafrost and viable *Bacillus anthracis* spores could have become exposed to the surface; the monthly average air temperatures in June, July, and August 2016 were 20–100% higher than those of the previous 30-year period, while the maximum air temperatures were 16–75% higher. Using the projected climate data for 2081–2100 according to the “worst case” RCP8.5 scenario, we demonstrated that the yearly air temperature may average above 0°C across the entire Yamal Peninsula, while the yearly number of days with a mean temperature above 0°C may rise by 49 ± 6 days, which would provide conditions for reactivation of soil anthrax reservoirs. Our results showed that the outbreak of anthrax occurred under conditions of a significant increase in air temperature in the study area, underlined the importance of vaccination for controlling the epidemic process, and demonstrated the effectiveness of monitoring studies using the IHA diagnostic kit for detecting erythrocyte anthrax antigens.

## Introduction

Anthrax is a bacterial disease affecting humans and other mammals, caused by the gram-positive, spore-forming, rod-shaped bacterium *Bacillus anthracis*. The main feature of this microorganism, which largely determines its epidemiological potential and population structure, is the ability to form endospores that are extremely resistant to unfavorable environmental conditions and are able to remain viable for a long time (1–3). Anthrax spores persist in the soil for a long time, and not only survive, but also become part of the soil biocenosis due to a combination of natural conditions. These include the structure of the natural soil cover, the chemical properties of the soil and its layers, and alternating floods and droughts, which increase leaching from the soil, drying, and dispersion of spores. The survival time of spores is unpredictable, which creates favorable conditions for the formation of natural foci (3–5).

The zoonotic potential of anthrax is well-known worldwide. Sheep, goats, cattle, buffaloes, horses, deer, donkeys, and other animals, including wild species, are known to be highly susceptible to anthrax, while pigs are less susceptible. Under natural conditions, rodents can also contract the disease (5). Currently, anthrax cases are extremely rare in most European countries, but the disease remains endemic in Russia, where it causes sporadic cases in animals and rare cases in the human population. The epidemic process usually involves grazing livestock becoming infected via the alimentary route, by ingesting *B. anthracis* spores while eating soil-contaminated plants or by drinking from water sources with a high concentration of spores (3, 4, 6, 7). The epidemiology of anthrax is characterized by summer–autumn seasonality, caused by grazing of animals on pastures that usually have sparse and dry grass. In the winter–spring (stall) period, infection is associated with the use of infected feed.

The prerequisites for the functioning of the anthrax biosystem are a constantly high level of soil contamination by *B. anthracis* spores, free grazing, and transhumance and semi-nomadic animal pasturing practice, which is typical for reindeer husbandry in the Far North of Russia (3). In Russia, the presence of large territories inhabited by populations of wild animals and livestock creates favorable conditions for outbreaks of epidemic diseases, and the low population density in most of the country makes it difficult to implement anti-epidemic measures and record the burial sites of animals that have died of anthrax correctly. Historical anthrax burials are often undocumented, and sometimes corpses are not buried properly. These burial grounds, as well as entire territories of historical epidemics, may be involved in economic activities, which may lead to new disease outbreaks given the marked preservation of spores in a cold climate.

Of particular interest in this regard is the tundra zone of Russia, located between 55 and 68 degrees North. The 1941 penultimate outbreak of anthrax in the Yamal Peninsula reindeer population resulted in the death of 6,700 reindeer. The last outbreak in 2016 killed more than 2,000 reindeer and led to the hospitalization of 90 local residents, as well as the death of one child (8–10).

Currently, in the northern regions of Russia, there is a risk of occurrence (revival) of soil foci of anthrax due the Yana, Indigirka, and Kolyma rivers flooding pastures and settlements, as well as large-scale earthworks (mining of diamonds, gold, oil, and gas; other types of subsurface use). This requires permanent preventive measures among livestock animals regardless of the current status of known anthrax foci, particularly in conditions where climate changes affect the habitat of macro- and microorganisms.

One important component of such preventive measures is the annual vaccination of susceptible animals, which is aimed at generating herd immunity against the disease in the population of livestock animals, including reindeer. The effectiveness of active immunization is monitored by evaluating the titer of anthrax antibodies (10–14). In 2007, the vaccination of reindeer in the Yamal Peninsula was canceled, which may have led to an increase in population susceptibility. In response to the 2016 outbreak, total vaccination of all susceptible animals was resumed.

The aims of the present study were (1) to evaluate the effectiveness of reindeer vaccination against anthrax performed as a response to the 2016 outbreak in the Yamal Peninsula, (2) to explore climate conditions in summer 2016 and their potential role in that outbreak, and (3) to assess the expected climate change on the Yamal Peninsula and its potential influence on the risk of anthrax resurgence.

## Materials and Methods

An ethical review was not required for this study, according to local and national legislation, because this work does not contain any experiments on animals. Blood samples for the study of anthrax immunity were collected by certified veterinarians using standard procedures to avoid the suffering of animals and followed the guidelines of the State Veterinary Service of the Russian Federation.

### Study Area

**Figure 1:**
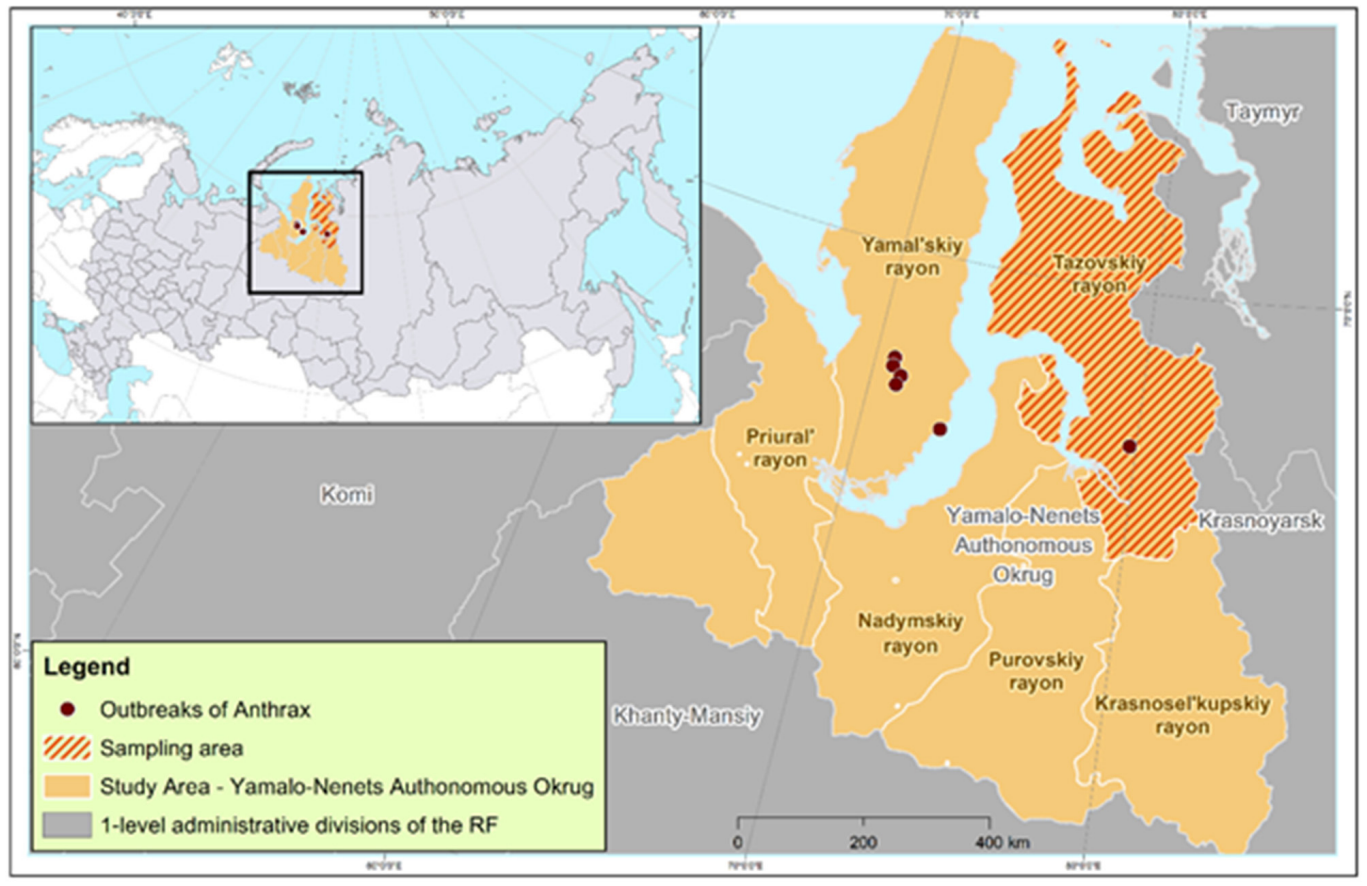
Study area, anthrax outbreaks in 2016, and the area of anthrax immunity monitoring in reindeer.

Our study assessed the anthrax epidemic situation in the Yamalo-Nenets Autonomous Okrug (YaNAO), which is one of the 85 regions included in the Russian Federation. The YaNAO is located in the Arctic zone of Russia, partly on the Yamal Peninsula. Most of its territory is located beyond the Arctic Circle (Figure 1). The area comprises 769,250 km^2^, and has a population density of about 0.7 persons/km^2^. The region is characterized by permafrost, a large number of rivers and lakes, and Arctic and subarctic climates in most of the territory. The total reindeer population in the YaNAO is estimated to be more than 700,000 head (15).

### Data Sources and Methods

#### Anthrax Data

The epidemiological analysis of the 2016 anthrax outbreak in reindeer in the YaNAO was based on official data by the Federal Service for Veterinary and Phytosanitary Surveillance (“Rosselkhoznadzor,” https://fsvps.gov.ru/fsvps/iac/rf/operative-messages.html) that were notified by Russia to OIE (https://wahis.oie.int/#/dashboards/country-or-disease-dashboard). The data contained information on the infected species, exact location of the outbreaks, and the number of infected and dead animals. This information was represented as a shapefile to enable mapping and spatial analysis in a geographic information system (GIS).

#### Climate Data

Meteorological data for the assessment of monthly mean and maximum air temperatures in the study area (the Tazovsky district of the YaNAO) from 1987–2016 were extracted from the official website of the Federal Service for Hydrometeorology and Environmental Monitoring (16) and from the Weather Archive web portal (http://pogoda-service.ru/archive_gsod.php) for the stationary weather station Antipayuta (synoptic index 23058; geographical coordinates 69°6′13^′′^N, 76°51′28^′′^E). We compared monthly mean and maximum air temperatures in May, June, July, and August for 2016 (the 2 months with recorded anthrax outbreaks and the two preceding months) with corresponding indicators for the entire 30-year period. Assessment of the statistical significance of differences between the temperature ranges was performed using the two-sample *t*-test with unequal variances.

The expected future change in the study area climate regime was assessed by comparing the average annual air temperature and the average annual number of days with an air temperature above 0°C for the current climate and for the projected climate for the period 2081–2100. To quantify the expected changes in both indicators, we averaged the calculated differences over the whole study area.

As a “current” climate, we used the indicators obtained by averaging the daily air temperature observations at meteorological stations in the Arctic zone of Russia from 1981–2015 (17), which were interpolated using a kriging^1^ (18–20) GIS technique with a spatial resolution of 1 km^2^.

The projected indicators for the period 2081–2100 were calculated by an ensemble of 13 climate models included in the international Coupled Model Intercomparison Project 5 (21) according to the RCP8.5 experimental scenario (22). The RCP8.5 scenario accounts for a high level of greenhouse gas-related climate forcing and assumes “no change” in humans' current behavior regarding anthropogenic atmosphere emissions. This scenario provides the most statistically significant models' response to climate forcing and may be used as a “worst case,” but still plausible, climate change scenario.

Climate data processing and visualization of results were performed with ArcMap 10.8.1 software including the Spatial Analyst extension (Esri, USA).

### Study on Post-vaccine Immunity

Reindeer were immunized in the Tazovsky district of the YaNAO in September and October 2016 with a dry live vaccine against animal anthrax from the strain 55-VNIVVIM, which was produced by the Oryol Biological Factory (Oryol, Russia) according to the instructions (http://www.biofabrika.com/catalog.html?cid=13). About 15,000 head were vaccinated.

Blood samples were then taken by veterinarians from 913 vaccinated reindeer in July 2017 within the framework of standard veterinary procedures. Blood sampling was performed from the jugular vein and samples were collected in disposable sterile test tubes. Blood serum was obtained by settling the blood, followed by aliquoting 1–2 ml of serum into Eppendorf test tubes. The blood serum was stabilized with boric acid.

Detection of specific anthrax antibodies in the blood serum of animals was performed with the indirect hemagglutination (IHA) test reaction with an anti-anthrax erythrocyte diagnostic kit. Due to the lack of commercial test systems in Russia (14), we produced an antigenic erythrocyte diagnostic kit in accordance with the “Temporary instruction for the manufacture and control of antigenic erythrocyte diagnostic kit for the indirect hemagglutination test reaction (IHA test reaction) (1979)” (23). The method for developing the kit was obtained from the State Scientific Institution for Veterinary Virology and Microbiology of the Russian Agricultural Academy, and the effectiveness of the kit has been confirmed in similar studies (14, 24, 25).

The IHA test reaction was carried out in accordance with the generally accepted procedure (26). The initial blood serum was diluted in a ratio of 1:40 with saline solution (taken as the first serum dilution), after which double dilutions were made and assayed using the erythrocyte diagnostic kit. The preliminary registration of the reaction was carried out after 2–3 h, and the final one after 18 h. The reaction result was assessed visually according to the following scheme:

++++: erythrocytes cover the entire bottom of the well in an even layer; sometimes, an “umbrella” pattern with uneven edges is observed;
+++: red blood cells cover 3/4 of the bottom of the well, and the “umbrella” is smaller;
++: the “umbrella” is small and is located in the very center of the well;
+: in the center of a small “umbrella,” the erythrocyte sediment is clearly visible (“buttons”).
–: negative reaction, erythrocytes settle on the bottom of the well in the form of a “button” or a small ring with even, well-defined edges.

The reaction results were considered positive when erythrocyte antigen agglutination was detected, starting with a dilution of 1:80 and higher by 3–4 crosses.

To assess the proportion of the whole reindeer population with specific immunity, the testing was considered as a hypergeometric process, where *n* samples were taken from a population *M* with a protection level *p*, of which *s* were positive (i.e., with a protective titer detected above 1:80). Since the sample size *n* was much lower than the whole population *M*, a binomial approximation of the hypergeometric process was used, and the expected protection level *p* was calculated using the beta distribution: *p* = Beta (*s* + 1; *n* – *s* + 1) (27). The calculations were performed using 10,000 Monte Carlo simulations in the Microsoft Excel add-in @RISK v.4.5 (www.palisade.com).

## Results

### YaNAO Anthrax Outbreak Descriptive Analysis

The anthrax epidemic in the Russian Arctic occurred in July–August 2016 in three territories: the area of Lake Pisoto, the Novoportovskaya tundra, and the area of the Evayakha River. The regions are located at a distance of up to 250 km from each other, and they include two water barriers: the Ob Bay (width: 30–80 km) and the mouth of the Taz River (average width of 25 km). The boundaries of six outbreaks were determined (Figure 1), and the susceptible reindeer population was estimated as 41,001 head. Out of the total number of animals, 2,650 (6.46%) animals developed clinical signs, of which 2,350 died (case fatality rate of 88.67%). The outbreak was characterized by the involvement of the human population in the epidemic process, which was manifested by the infection of 36 people with one estimated fatal outcome (28).

An analysis of official reports and directives showed that animals had not been vaccinated in the region of anthrax origin since 2007.

### Climate Data Analysis

The analysis of the mean and maximum monthly air temperatures in the spring and summer months of 2016 demonstrated no statistically significant differences between temperatures in May with corresponding values from 1987–2016, while mean temperatures for June, July, and August were found to be 16–100% higher in 2016 (Table 1). In general, a rise in summer air temperatures was observed in 2007–2016, reaching peak values of 15.4, 22.1, and 15.0°C in June, July, and August of 2016, respectively.

**Table 1:** The results of air temperature comparison in May, June, July, and August 2016 with corresponding indicators for 1987–2016 based on weather station Antipayuta data.

		**Mean** **±** **Standard deviation**, ^****°****^**C**		**Difference of mean, ^**°**^C**	**Difference of mean, %**	***t*-test *p*-value**
		**1987–2016**	**2016**			
Monthly mean air temperature	May	−4.4 ± 4.7	−4.4 ± 5.1	0	0	>0.1
	June	5.5 ± 5.4	11.0 ± 5.6	5.5	100%	<0.001
	July	13.0 ± 4.8	17.4 ± 4.7	4.4	34%	<0.001
	August	9.5 ± 3.3	11.5 ± 3.2	2.0	21%	<0.001
Monthly maximum air temperature	May	−1.9 ± 4.3	−2.2 ± 4.5	−0.3	−16%	>0.1
	June	8.8 ± 6.6	15.4 ± 6.6	6.6	75%	<0.001
	July	17.0 ± 5.5	22.1 ± 5.4	5.1	30%	<0.001
	August	12.9 ± 3.8	15.0 ± 3.6	2.1	16%	<0.001

**Figure 2:**
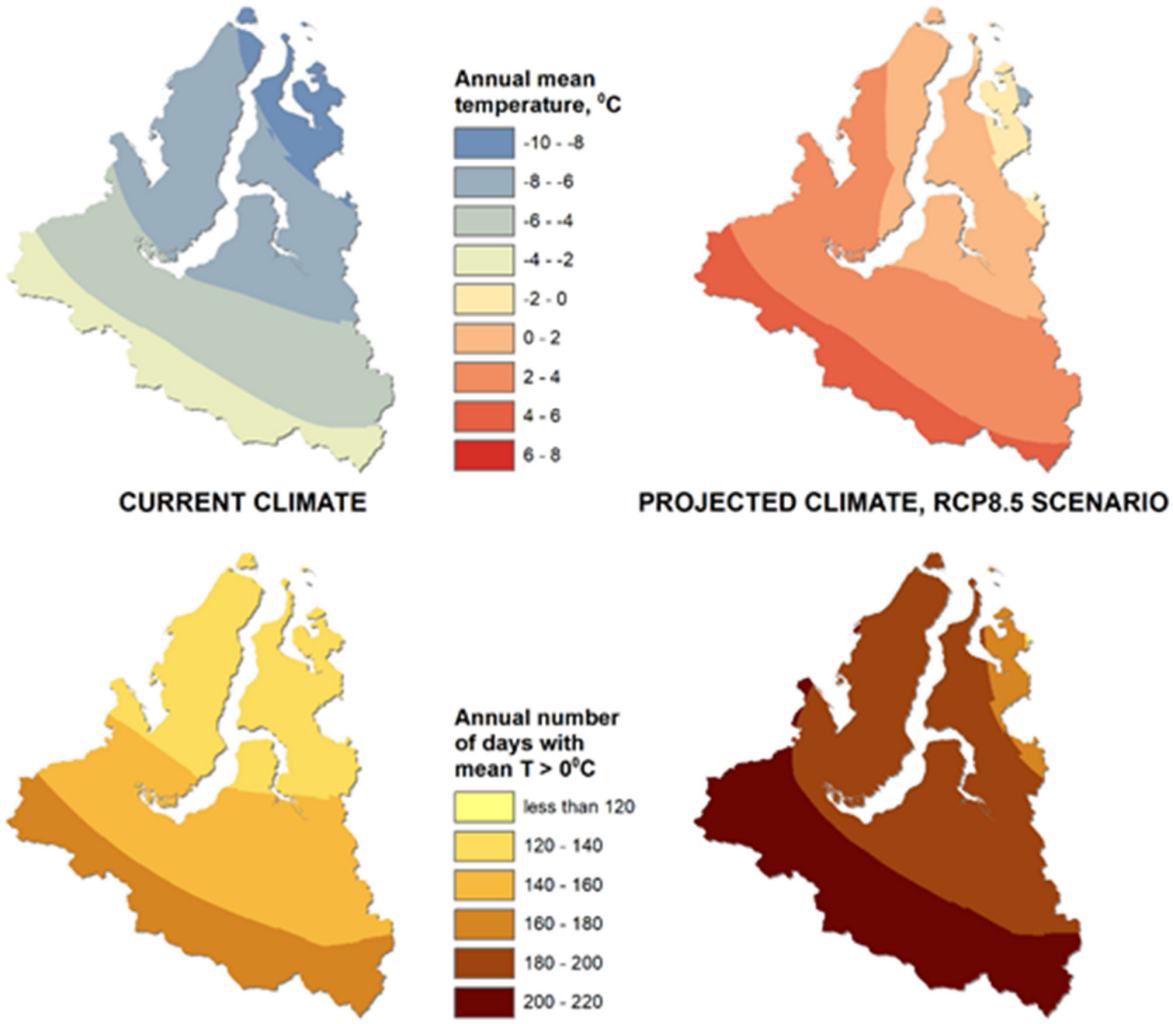
Results of modeling the average annual air temperature (top) and the number of days per year with an air temperature above 0°C (bottom) on the territory of the Yamal-Nenets Autonomous Okrug for the current climate and for the projected climate (2081–2100) according to the RCP8.5 scenario.

Modeling of the current and projected climate indicators showed that, in the “worst case” scenario of warming by 2100, the average annual air temperature within the study area is expected to increase by 8.3 ± 0.5°C and will be positive in most parts of the YaNAO, while the yearly number of days with air temperatures above 0°C may rise by 49 ± 6 days (Figure 2). Such climate changes may result in further thawing of permafrost over the whole study area and could aggravate the situation with thawing of the soil containing anthrax spores.

### Vaccination Effectiveness Assessment

In 2007, the government discontinued routine vaccination of reindeer on the Yamal Peninsula, which was considered free of anthrax. Given the time span between the last vaccination in 2007 and the outbreak in 2016, it is likely that the vast majority of animals were not vaccinated prior to exposure to the anthrax bacterium in 2016. Such a naïve population is fertile ground for the spread of an infectious agent, as all or most animals would not have had immunity to anthrax.

**Figure 3:**
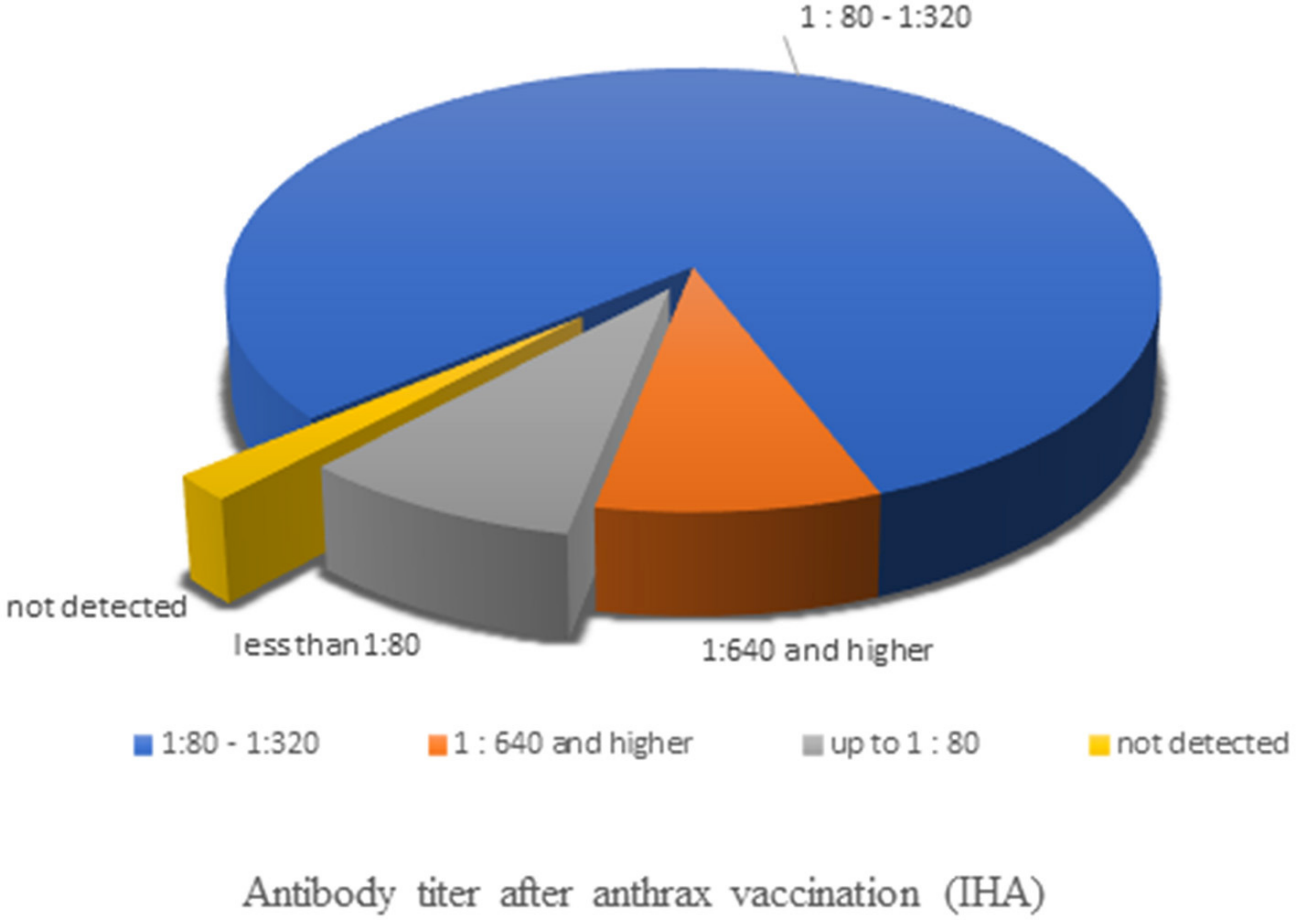
The results of blood serum sample analysis in the indirect hemagglutination assay.

The strength of immunity against anthrax after resumption of the vaccine in 2016 was determined in the IHA blood serum results (Figure 3). Screening of 913 samples of reindeer blood sera for anthrax antibodies by this assay showed that 1.97% of samples (18 head) did not contain anthrax antibodies. In 8.87% of samples (81 head), anthrax antibodies were detected at a titer of 1:80, which is lower than the protective level. In 89.16% of the samples (814 head), the level of specific antibodies at 9 months after vaccination was ≥1:80, of which 9.0% of the samples (82 head) had a serum anthrax antibody titer of ≥1:640. The data obtained indicate the creation of an immune proportion of 89% (95% confidence interval: 87–91%) of the reindeer population in the Tazovsky district of the YaNAO.

## Discussion

In this study, we evaluated the effectiveness of the reindeer vaccination against anthrax performed as a response to the 2016 outbreak in the Yamal Peninsula, explored the contribution of climate conditions in the summer of 2016 to that outbreak, and assessed the expected climate change in this region and its potential effect on the anthrax resurgence risk. We found that the vaccination led to a protection level of 89% of the whole reindeer population. Moreover, an abnormally high ambient temperature in summer 2016 contributed to the thawing of permafrost and the reactivation of soil reservoirs of *B. anthracis*. Using the projected climate data for 2081–2100 according to the “worst case” RCP8.5 scenario, we demonstrated that the expected climate change would provide conditions for reactivation of soil anthrax reservoirs.

Vaccination-based anthrax prevention in many developed countries has led to a decrease in the number of registered disease cases (29–36). However, the risk of anthrax remains high in sub-Saharan Africa, Southeast Asia, and parts of Russia and the former Soviet Union countries (37–40). Serological testing is necessary to determine the effectiveness of vaccination programs and obtain epidemiological information in anthrax-endemic areas. In Russia, the effectiveness of the vaccination of reindeer against anthrax has not previously been evaluated; therefore, no data exist on the levels of antibodies in reindeer during 2007–2017. To the best of our knowledge, this research is the first in the field to investigate this in reindeer specifically. However, data from studies on the effectiveness of anthrax vaccination in livestock animals demonstrated high levels of antibodies in cows and sheep for about 1.5 years (41). This suggests that at the time of the 2016 outbreak, there could be no immunity among reindeer. In this study, we demonstrated the usefulness of anthrax vaccination by screening reindeer herds in the Tazovsky district of the YaNAO for anthrax antibodies using an IHA. In 89.16% of the samples, the level of specific antibodies by 9 months after vaccination was ≥1:80, of which 9.0% of the samples had a serum anthrax antibody titer of ≥1:640. This indicates the establishment of immunity in 89% of the reindeer population. No new anthrax outbreaks have been observed in the YaNAO since 2016. Based on the concept of herd immunity, the number of immune individuals in the population needed to prevent the spread of infection should be at least 1 − 1/R_0_, where R_0_ is the basic reproductive ratio of the disease (42). Accepting the R_0_ value for anthrax as 1.251–1.292 according to previous studies (43, 44), we obtained a minimum herd immunity threshold of 20–23%. Thus, the immune proportion achieved as a result of vaccination can be considered sufficient to prevent the spread of anthrax in the reindeer population. The absence or low levels of anthrax antibodies in a part of the population may indicate omission of anthrax vaccinations or violations of vaccination rules (vaccination of pregnant, weakened, and emaciated animals, etc.), or it could be related to the characteristics of some individuals.

Currently, climate change is more pronounced in the Russian Arctic than in any other part of the country. The fastest rates of environmental changes on the Earth are observed in this region, including a decrease in the extent of sea ice, melting of glaciers and ice sheets, lengthening of the growing season, melting of permafrost, and strengthening of the hydrological cycle. The average annual air temperature in the Russian North increased by 1.28°C from 1905–2000 (40). The global temperature of the Earth's surface warmed by 0.89°C from 1901–2012 (45). Permafrost is any ground that remains completely frozen below 0°C for at least 2 consecutive years (46). Permafrost may reach a base depth of more than 1,000 m and remain frozen for thousands of years.

In permafrost conditions, microorganisms are well-preserved and the permafrost acts as an accumulator of microbiota. The preservation of *B. anthracis* spores in permafrost conditions is not doubted (10). Permafrost temperatures increased by 0.29 ± 0.12°C worldwide following the increase in air temperature in the Arctic (47). Permafrost in the Russian Arctic has thus become a reservoir for anthrax that can preserve viable spores for a long time. The high air temperature in the studied region could lead to the thawing of permafrost and the opening of soil anthrax reservoirs, as well as an increase in the number of blood-sucking insects that may carry the anthrax pathogen (10, 48). Consequently, global warming in the Arctic could become one of the risk factors for the occurrence and spread of anthrax due to thawing of the permafrost (49, 50). Under changed climatic and soil-hydrological conditions, these spores could have appeared on the soil surface and consequently infected animals. Our modeling has shown that in the case of an unfavorable climate change scenario, the average annual air temperature on the territory of the YaNAO will exceed 0°C, creating conditions for widespread permafrost thawing.

The second risk factor for epidemic anthrax is the discontinuation of vaccination. The Yamal tundra is the largest center of reindeer husbandry in the Arctic; for example, 254,000 head of reindeer were grazed in the Yamal district of YaNAO in 2015 (51). Thus, the epidemic and economic risks are very high in this region. Retrospective analysis of climate data and modeling indicated the effectiveness of anthrax prevention by annual vaccination of reindeer.

To assess the extent of coverage of the animal population and the effectiveness of vaccination, serological monitoring should be carried out, including the use of the IHA diagnostic kit for detecting erythrocyte anthrax antigens.

## Conclusions

This study showed that the factors that caused the 2016 anthrax epidemic in the Russian Arctic may include the increase in summer air temperatures, the climate change-induced thawing of permafrost, and the discontinuation of vaccination of reindeer against anthrax. This led to the creation of an anthrax epidemic chain. Moreover, the expected climate change according to the most unfavorable scenario may contribute to marked warming in the YaNAO territory, creating conditions for widespread thawing of permafrost and reactivation of anthrax foci in soil. The level of post-vaccination immunity in reindeer detected in our study can be considered sufficient to prevent anthrax outbreaks, including under conditions of thawing permafrost in the Russian Arctic. In the period 2017–2020, no new outbreaks of anthrax were recorded in the Yamal Peninsula, which can be ascribed to the traditional practice of annual vaccination against anthrax. Moreover, the IHA developed here is an effective tool that will be useful for monitoring the effectiveness of reindeer vaccination in the context of an increasing epidemic risk of anthrax in the Arctic.

## Data Availability Statement

The data analyzed in this study is subject to the following licenses/restrictions: The datasets of climatic variables as well as reindeer blood samples data cannot be publicly shared due to confidentiality. They can be obtained on reasonable request from the corresponding authors. Requests to access these datasets should be directed to Elena A. Liskova, liskovaea@mail.ru.

## Author Contributions

EL, AB, and FK: conceptualization, validation, and formal analysis. IE, EL, AB, GS, and SM: methodology. FK and OZ: software. IR, OZ, and NT: investigation. NG, OB, and GS: resources. EL and AB: data curation and supervision. EL, IR, OZ, and AB: writing—original draft preparation. EL, YS, FK, and AB: writing—review and editing. OZ and FK: visualization. IE and II: project administration. All authors have read and agree to the published version of the manuscript.

## Conflict of Interest

The authors declare that the research was conducted in the absence of any commercial or financial relationships that could be construed as a potential conflict of interest.

## Footnotes

^1^Kriging is a procedure of spatial interpolation that allows obtaining a predictive geostatistical surface based on a sparse set of sample data (18, 19). This is a most widely used geostatistical technique to convert point weather station climate observations into a smooth raster surface (20).
